# Comparison of Digital Interactive Case-Based Educational Resource with Virtual Role Play in Dental Undergraduates in Clinical Oral Medicine/Oral Pathology Education

**DOI:** 10.3390/healthcare10091767

**Published:** 2022-09-14

**Authors:** Wen Xi Tee, Siew Huey Tan, Fareeza Marican, Preena Sidhu, Swarna Yerebairapura Math, Divya Gopinath

**Affiliations:** 1 School of Dentistry, International Medical University, Kuala Lumpur 57000, Malaysia; 2 E-Learning Resources Department, International Medical University, Kuala Lumpur 57000, Malaysia; 3 Restorative Dentistry, International Medical University, Kuala Lumpur 57000, Malaysia; 4 Clinical Oral Health Sciences, International Medical University, Kuala Lumpur 57000, Malaysia; 5 Centre for Transdisciplinary Research, Saveetha Dental College, Saveetha Institute of Medical and Technical Science, Chennai 600077, India

**Keywords:** dental education, interactive resource, role play, virtual, simulation

## Abstract

Case-based learning has always been a key element of dental education and the incorporation of technology into the concept became increasingly important during the COVID-19 pandemic. This study aimed to compare the effectiveness of a virtual interactive case resource in oral medicine/oral pathology, Virtual Oral Medicine Clinic (VOMC), with the virtual role play among third-year dental undergraduates. Fifty-one students were randomly assigned into two groups and the control group was subjected to a role play activity, whereas the experimental group was provided with VOMC. Both groups were assessed with an objective structured clinical examination (OSCE) before and after the intervention. Students’ self-perceived usefulness of the interventions was evaluated by a questionnaire and randomly selected students were invited for focus group discussions. Data were analysed using the Wilcoxon signed-rank test and the Mann–Whitney U test. Descriptive statistics were used to analyse student responses. Students in both groups demonstrated significant improvement (*p* < 0.001) in the post-test compared to the pre-test. Students in the experimental group demonstrated higher overall scores (*p* < 0.001) when compared to the control group. Though both methods were received favourably by the students, role play was more positively perceived when compared to digital resource. Though VOMC was shown to improve student scores, the perception of VOMC was not quantitatively superior to the role play activity. Hence VOMC can be recommended as an adjunct tool to enhance learning in oral medicine in undergraduate dental students.

## 1. Introduction

The COVID-19 pandemic has had a significant impact on every aspect of life. More than 94% of students in the world were affected by the closures of schools, institutions, and other learning settings [1]. Schools and universities have put much effort into innovating and implementing alternative educational practices. This pandemic not only created the demand for but also presented an opportunity to expedite digital transformation in medical education. This could have a favourable impact on dentistry education in the future, even beyond COVID-19.

Digital education (often referred to as e-learning) is “the act of teaching and learning utilizing digital technologies” [2]. The advantages of digital learning include accessibility, ease of use, flexibility of time, and superior quality of images [3]. Although almost every student agreed that digital learning was a good option during the COVID-19 pandemic, many of them did not feel well prepared for the practical aspect of the curriculum via online education [4]. Interactive computer simulations of real-life clinical scenarios, either termed as virtual patient cases or virtual interactive cases, for the purpose of health professionals’ training, education, or assessment have been increasingly used recently [5,6,7]. Though this broad definition embraces a variety of methods that use various technologies and focus on various learning needs, these resources are postulated to primarily address clinical reasoning. The educational use of real-life clinical scenarios on a digital resource can be explained based on experiential learning theory [8]. Exposing the learners to simulated clinical experiences helps in scaffolding the mechanisms for information gathering, clinical reasoning, and decision-making in a safe environment [9,10].

Virtual interactive cases are designed in such a way that the student collects data, typically through menus of potential lab tests, physical exams, and history inquiries, and then diagnoses and/or manages the patient. The information is not “cued,” i.e., the programme structure does not indicate what the pupil should do next with the knowledge. This kind of design makes it simple to develop templates, which could lower the cost of running several simulations. Exposing the learner to numerous simulated clinical scenarios encourages learning clinical reasoning and helps in acquainting the learners with a regular set of common clinical conditions in the population that are not prevalent in individual teaching institutions. [9,10]. We developed an interactive digital case-based educational resource in oral diagnosis, Virtual Oral Medicine Clinic (VOMC), for third-year dental undergraduates that illustrates dentist–virtual patient interaction pertaining to oral medicine/oral pathology clinical cases. To the best of our knowledge, there are no studies assessing the impact of case-based e-learning digital resource intervention in undergraduate education on oral medicine/oral pathology. Therefore, this study aimed to evaluate the effect of VOMC on cognitive skills (diagnosis and decision making), communication, and professionalism in third-year dental undergraduates and to compare it with conventional role play in an online environment (virtual role play). Additional data on attitudes and self-perceived usefulness towards their learning experience were also collected.

## 2. Materials and Methods

### 2.1. Study Design

A randomized study was carried out on third-year dental undergraduates at International Medical University (IMU), Kuala Lumpur, Malaysia. The convenience sample included 51 students randomly divided into two groups—a control group (27) and an experimental group (24). A briefing session was held to inform about the study design and intervention of virtual role play and VOMC. Participants in the control group were exposed to virtual role play activities, while participants in the experimental group were exposed to an interactive digital oral medicine case-based educational resource for four weeks. A pre-test OSCE was conducted before the intervention and followed by the post-test OSCEs. The study was approved by IMU-JC (BDS I-01/2021(04)). Before the commencement of the study, written informed consent was obtained from each participant.

### 2.2. Selection of Case Scenarios

Five case scenarios were selected based on the following topics: oral ulcers, odontogenic pain and swelling, intraoral white patches, orofacial pain, and oral manifestations of systemic disease. These topics were aligned to the selected learning outcomes of the third-year oral diagnostic sciences 1 module in the dental curriculum at the university. The VOMC resource and the role play scripts were developed based on these selected case scenarios.

### 2.3. Virtual Oral Medicine Clinic (VOMC)

An interactive digital oral medicine case-based educational resource, Virtual Oral Medicine Clinic (VOMC), was designed and developed by IMU eLearning Department based on the instructional design model of ADDIE (Analysis, Design, Development, Implementation, Evaluation) as a framework for designing an educational e-learning content. The e-learning tool that was used to develop the interactive content was Articulate Storyline 360 and Adobe Illustrator.

**Figure 1:**
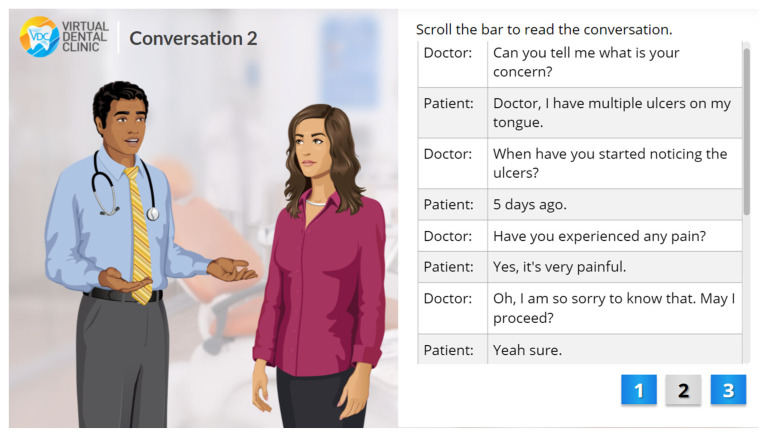
Dentist–patient interaction in history taking.

**Figure 2:**
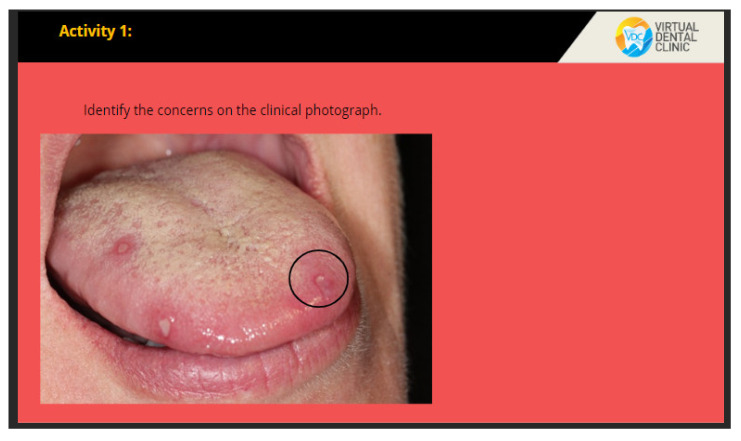
Example of the interactive activity where students can click and identify the areas of concern.

**Figure 3:**
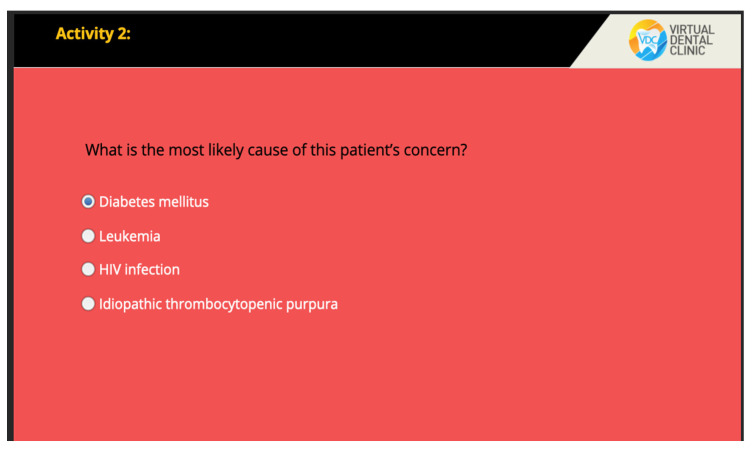
Self-assessment tool.

The VOMC showcases dentist-patient interaction with the following scenes: history taking, clinical examinations, investigations, treatment planning, and self-assessment tool. The interface illustrating the dentist-patient interaction is shown in Figure 1. The users are prompted to interact with components including clicking on the area of concern on clinical images or defining the extent of radiographic changes as well as identifying histopathological components on the screen (Figure 2). Moreover, there is a self-assessment tool consisting of 5 multiple-choice questions in the digital resource (Figure 3). After the learner’s attempt, evidence-based answers, feedback, and explanations of the questions were provided. Students were allowed to access the digital resource repeatedly within the intervention of four weeks.

### 2.4. Virtual Role Play Activity

Five virtual role play sessions involving the same case scenarios were scheduled for the control group within a time frame of four weeks. During each session, the patient’s script was provided to the facilitator (faculty) who enacted as the patient while one of the students in the control groups enacted as a clinician. After students completed the history taking, the clinical images of oral lesions were provided to the students. After the student mentioned the investigations, the findings of the investigation were provided to them. At the end of role playing, students presented complete history, provisional diagnosis, differential diagnosis, investigations, and management of the given case to the facilitator.

### 2.5. Learning Evaluation

The effect of interventions was evaluated by conducting a virtual objective structured clinical examination (OSCE). There were five OSCE cases aligned to the same learning outcomes. Students in both groups were assessed in two phases: before the intervention (pre-test), and after the intervention (post-test). The examiner acted as the simulated patient. The clinical images, radiographic images, or investigation results were provided for the patients as routine OSCEs to reach a provisional diagnosis and management plan. Students were divided into four groups and four different trained examiners were involved in the process. All the examiners were well-accustomed to the OSCE process and were provided with the simulated patient script as well as answer rubrics. For each OSCE the students were assessed using the following domains on a global rating scale (bad, poor, unsatisfactory, satisfactory, good) on four domains, namely, (1) Professionalism and Patient Safety, (2) Communication Skills, (3) Diagnostic Skills, and (4) Decision making in Clinical Management. This global rating scale is commonly used for summative OSCE in our school. The school has standard checklists that are critically reviewed, and standards are set for performance expectations. The same examiners assessed the students in pre-tests and post-tests so that any bias could be avoided. Professionalism was assessed by the way the student met and greeted the patients, the way the student tried to build trust and confidentiality in the conversation and the empathy for the patient’s voice and their conversation.

Students were reassured that the test results would not be used in their summative assessments.

### 2.6. Student’s Feedback

#### 2.6.1. Online Questionnaire

The perceived usefulness of role play and VOMC was collected using an online questionnaire on a four-point Likert scale (strongly agree, agree, disagree, strongly disagree) adopted from previous studies [11,12]. The questionnaire was divided into three domains: (1) diagnostic skills, (2) communication skills, and (3) impact on learning. The diagnostic and communication skills contained 5 questions each, and the impact on learning included 3 questions.

#### 2.6.2. Focus Group Discussion

Focus group discussions were used to assess students’ perceptions of role play and the VOMC. The discussion was broadly classified into the following themes: comparison of role play activity and VOMC, attitude towards learning diagnostic and communication skills through role play activity and VOMC, the usefulness of role play activity and VOMC in clinical practice and contentment of learning diagnostic as well as communication skills. Eight randomly selected students were invited to participate online. The focus group discussion was carried out in Microsoft teams and lasted for 30 min. The session consisted of one member of the study team as moderator and another as a note taker. The session was recorded, and the content was transcribed on the same day as the discussion.

### 2.7. Data Analysis

Quantitative data from pre- and post-test OSCE scores were tabulated and analysed using SPSS software version 28.0. Descriptive statistics were determined for each variable. Shapiro–Wilk test was conducted to determine the normality of data (*p* < 0.05). As the majority of the data were not normally distributed, non-parametric tests were utilized. A comparison of pre-test and post-test scores was made using Wilcoxon signed-rank test and the Mann–Whitney U test was conducted to compare the scores between control and experimental groups. A *p*-value of <0.05 was considered statistically significant. Frequency distributions of responses on the perceived usefulness of interventions were calculated. Mann–Whitney U test was conducted to compare the questionnaire results between control and experimental groups. A *p*-value of < 0.05 was considered statistically significant. The focus group discussion data were analysed qualitatively based on themes.

## 3. Results

A total of 51 students, enrolled in the Bachelor of Dental Surgery (3rd year) at International Medical University, participated in the study. Their age ranged from 21 to 30 years (mean age = 22.94 years old ± 1.256). There were more female (74.5%, *n* = 38) than male participants (25.5%, *n* = 13).

### 3.1. Learning Evaluation

Since the sample size of the study was small, determining the distribution of variables was critical in choosing the suitable statistical method to use. The Shapiro-Wilk test showed that the distribution of variables deviated significantly from normality for professionalism and patient safety (*p* < 0.017) and diagnostic skills (*p* < 0.029). Based on this result, non-parametric methods were used. The means and standard deviations of the variables in both groups are shown in Table 1. Students in both groups demonstrated greater mean scores in post-test compared to pre-test, showing statistically significant differences, *p* < 0.001, on all four domains. The total mean scores of post-test in both groups were significantly higher compared to pre-tests (*p* < 0.001). The comparisons of mean scores between the control and experimental groups are presented in Table 2. Overall, students in the experimental group demonstrated greater mean scores compared to the control group in the post-test (*p* < 0.001). Across the domains, the mean scores for the experimental group were more than the control group (Table 2).

### 3.2. Student’s Feedback

#### 3.2.1. Feedback Questionnaire

A feedback questionnaire was used to evaluate participants’ self-perceived usefulness of the intervention. Table 3 and Table 4 show the results in the mean score and frequency distribution of the control group (*n* = 17) and experimental group (*n* = 20). Generally, the advantages of both interventions in improving diagnostic and communication skills were well perceived by most of the students, with control group students showing higher overall agreement than experimental group students.

All control group students agreed that role play helped them in diagnosing mucosal lesions and 94.1% agreed that it increased their confidence and helped them in formulating an appropriate management plan. In the experimental group, improvements in diagnostic skills, interpretation of variations in mucosal lesions, and management planning were agreed upon by 85%, 75%, and 90% of students respectively, while only 65% of them agreed that VOMC increased their confidence in the diagnosis of mucosal lesions. Participants in the control group showed 100% agreement that role plays improved their communication skills, made dentist-patient interaction more interesting and realistic, as well as reduced their anxiety when communicating with patients. In the experimental group, the majority of them agreed that VOMC improved their communication skills (85%), reduced their anxiety when communicating with patients (80%), and made them more confident in communication (80%). However, only 55% of students agreed that VOMC made dentist-patient interaction more interesting and realistic.

In the control group, every student agreed that a role play activity positively enhanced their learning experience and made them more concentrated on learning diagnostic and communication skills in oral medicine. Most of the participants in the experimental group agreed that VOMC positively enhanced their learning experience (90%). However, only 60% thought that VOMC helped them to be more concentrated in learning diagnostic and communication skills in oral medicine.

Statistically, the mean scores for the control group were more than the experimental group across the domains. (Table 5)

#### 3.2.2. Focus Group Discussion

The issues discussed in this focus group discussion were broadly classified into four themes:

(1) flexibility, (2) organization of content, (3) interactivity, and (4) element of personalization.

Flexibility: A few participants were more comfortable with the flexibility of VOMC. For instance, students can go through “VOMC at their convenience and as many times as they could compare to role play which is fixed”. They agreed that VOMC was a good additional learning resource as it provide a good framework for the new topic.

Organization of content: The students’ view was that VOMC is more organized in terms of sequence as compared to the role play activity, which is hard to follow the sequence and might miss out on some of the important points. One of the participants expressed that “VOMC is helpful in their OSCE exams as the doctor in VOMC explains everything in layman’s terms to the patient”.

Interactivity: Some of the students believed that VOMC is not interactive as compared to role play. They thought that VOMC is quite boring and long compared to role play. They also claimed that the tone in “VOMC is quite monotonal which is the least favourite aspect of VOMC”.

Element of personalization: Some students claimed that VOMC is more computerized as compared to role play and an element of personalization was missing. In role play, they felt the “feedback provided by the onsite supervisor is more personalized rather than autogenerated in the case of VOMC”.

## 4. Discussion

We evaluated the utility of a virtual interactive case resource, VOMC, and our results showed that VOMC is more effective than a role play activity in improving skills in cognitive domains tested among third-year dental undergraduates, namely, diagnosis and clinical management (decision making). Previous studies on dental undergraduates have reported that virtual education was more effective than traditional education in improving outcomes in the cognitive domain [13,14,15] or equally effective [16,17,18]. However, some of them utilized the same set of questions for pre-tests and post-tests [13,16]. Our study utilized different questions, although aligning with the same learning outcome. Moreover, most of these studies utilized single best answer questions, whereas we used OSCEs to compare the performance of the students pre- and post-intervention. Furthermore, as our data indicate, we also ensured the comparability between the two groups at baseline, which is quite crucial to ensure homogeneity before any intervention.

Apart from the cognitive domain, our results also highlighted that the VOMC is as effective as role play in improving communication skills among dental undergraduates. This is in agreement with a previous study where student-generated videos of clinical scenarios improved communication skills and collaborative learning skills [19]. Communication is an essential component in building a good dentist–patient relationship. Good clinical communication can address patients’ concerns, increase patient satisfaction, and provide a better dental experience [20]. Due to the importance of clinical communication skills in promoting the dentist–patient relationship, specific educational content for communication skills training has been developed in several dental schools in recent years. Traditionally, role play has been established as an effective method of training students to improve clinical communication skills. Role play is defined as an active learning activity in which participants act out a set of defined role behaviours or positions to acquire desired experiences [21]. It provides an opportunity for the student to have pre-clinical exposure to “real world” situations with positive attitudes and feelings [22]. Al-Khalifa and Nazir previously showed that role play evoked positive perceptions in improving students’ communication skills and professional behaviours [23]. Thus, regardless of the incorporation of technology into education, our results highlight that active learning strategies can create effective learning environments and can improve communication. Student engagement and active learning have always been the key concepts regarded as the basis of sound educational practices [24].

Evaluating professionalism as a part of the OSCE has been debated in the medical education literature [25,26]. OSCEs are widely-used, established tools for assessing history-taking skills, physical examination, and clinical decision-making skills; more recently, assessing professionalism in OSCEs has been introduced and is a common practice widely [25]. The students who were subjected to VOMC scored higher in professionalism. This might be because the dentist–patient interaction shown in the VOMC is a perfect model for them to copy and learn. Role modelling of medical teachers by students has been advocated as one of the most powerful methodologies of mentoring professionalism in medical education [27,28]. However, because of the complexity of the behaviour definition involved in assigning ratings to professionalism in doctor–patient encounters, it has been debated that there can be potential subjective variation based on examiners [24,25]. Hence it has been proposed to incorporate a special station dedicated to evaluating professionalism in OSCEs rather than incorporating this domain in every OSCE [24,25], which is the usual practice now. Future studies can incorporate a similar strategy to evaluate professionalism.

Although there was a significant improvement in the post-test scores for both the groups, it is necessary to note that participants’ self-appraisals of the learning experience were reported in favour of the role play. Previous studies have shown that motivation and active engagement in learning are important parts of role play [22,23]. Many students in the experimental group agreed that VOMC made dentist–patient interaction more interesting and realistic; however, they still preferred role play. Through our focus group discussion, we explored the reason for this student’s preference. Lack of personalization was identified as a significant disadvantage of VOMC by our study participants. Role play presented the convenience of having an active discussion with the facilitator, especially when acquiring knowledge regarding differential diagnoses beyond the scope of given case scenarios. Furthermore, a role play activity better-stimulated students’ critical thinking skills as the facilitator asked questions to all the students, encouraging them to brainstorm for answers. At the end of the role play activity, the facilitator gave personalized feedback on the performance of students, while students using VOMC were not able to review their performance in that detail. These factors might contribute to the higher perceptions of the usefulness of the role play activity compared to VOMC. Another concern was the use of a computer-generated monotonic voice-over in the digital educational resource. The monotonic voice may dampen the patient’s emotions, thus making it difficult to show empathy and build rapport with patients.

A study conducted by Wu et al. showed that students showed a higher positive perception of the use of virtual resources compared to role play because it enhanced their motivation and engagement in learning, as well as reduced their anxiety in oral communication [11]. However, in contrast, our present study showed that students in the control group demonstrated higher agreement on reducing anxiety through the role play activity. Our students considered role play as a safe learning environment where they got to practice as well as reinforce their skills in history taking, empathy, and communication skills pertaining to top principles of experiential learning theory. Additionally, students also highlighted the distractions while they were using their laptops or electronic devices unsupervised. It is important to consider that students’ attitudes, learning styles, and personality traits might also be considered significant contributing factors towards perceptions. [28] However if future research plans to closely approximate experiences gained from role play, methods for alleviating these concerns and incorporating the positive experiences into interactive digital resources need to be explored. With many medical and dental programs exploring alternatives to chairside clinical education, the authors believe this is a remarkably crucial area for further research. Further research should explore how to add digital resources to the traditional methods with the incorporation of the element of personalization and interactivity and how best they can be integrated into the existing dental education curriculum.

There are some limitations of this study that should be acknowledged. Firstly, the findings of this study may not be generalizable to students in other programs as it was conducted in one dental school during a single academic year. The study sample size of 51 participants could be considered a small sample. Though the examiners were briefed regarding the marking of the OSCE, the lack of calibration in a standardized format could have introduced potential bias in pre-test and post-test scores. Although four core themes and several subthemes emerged during the focus group discussion, the authors are aware that there must be other potential strengths and weaknesses of this resource as perceived by undergraduate dental students.

## 5. Conclusions

In conclusion, our study compared students’ learning experience of a newly developed interactive virtual case resource, VOMC, with traditional role play using quantitative and qualitative approaches. The results of our study imply that, while evolving digital tools can enhance students’ learning experience in oral medicine and diagnosis, students’ perception of an enhanced learning experience is based on direct interaction with facilitators as in role play. By considering the student perceptions, it would be appropriate to recommend the tool as an adjunct to the traditional role play methods as it would facilitate comprehension and a better understanding of the learning outcomes.

## Figures and Tables

**Table 1:** Comparisons between OSCE pre-test and OSCE post-test scores of each group.

		Control (*n* = 27)			Experimental (*n* = 24)		
		Mean	SD	*p*-Value	Mean	SD	*p*-Value
Professionalism and Patient Safety	Pre-Test	17.30	3.036	<0.001	17.83	3.130	<0.001
	Post-Test	19.81	3.465		23.17	2.444	
Communication Skills	Pre-Test	15.00	3.113	<0.001	16.54	2.553	<0.001
	Post-Test	20.48	2.779		23.08	2.185	
Diagnostic Skills	Pre-Test	14.89	3.309	<0.001	15.83	3.002	<0.001
	Post-Test	20.63	2.290		21.96	2.053	
Clinical Management (Decision making)	Pre-Test	14.33	3.174	<0.001	15.08	2.339	<0.001
	Post-Test	19.85	2.797		21.50	1.911	
Total	Pre-Test	61.52	10.885	<0.001	65.29	8.508	**<0.001**
	Post-Test	80.78	9.521		89.71	6.517	

**Table 2:** Comparisons between control and experimental groups.

		Pre-Test (51)			Post-Test (51)		
		Mean	SD	*p*-Value	Mean	SD	*p*-Value
Professionalism and Patient Safety	Control	17.30	3.036	0.456	19.81	3.465	<0.001
	Experimental	17.83	3.130		23.17	2.444	
Communication Skills	Control	15.00	3.113	0.019	20.48	2.779	0.001
	Experimental	16.54	2.553		23.08	2.185	
Diagnostic Skills	Control	14.89	3.309	0.216	20.63	2.290	0.027
	Experimental	15.83	3.002		21.96	2.053	
Clinical Management (Decision making)	Control	14.33	3.174	0.209	19.85	2.797	0.045
	Experimental	15.08	2.339		21.50	1.911	
Total	Control	61.52	10.885	**0.091**	80.78	9.521	**<0.001**
	Experimental	65.29	8.508		89.71	6.517	

**Table 3:** Distribution of students’ perceived usefulness of role play activity (*n* = 27).

Question: I Think to Role-Play Activity No of Respondents (27)	Mean (SD)	Strongly Agree (%)	Agree (%)	Disagree (%)	Strongly Disagree (%)	Overall Agreement (%)
Diagnostic Skills						
1. improves my diagnostic skills in Oral Medicine	3.8(0.4)	76.5	23.5	0	0	100
2. helps me to interpret variations in clinical presentation of oromucosal lesions/conditions	3.7(0.5)	70.6	29.4	0	0	100
3. helps me to diagnose oromucosal lesions/conditions	3.7(0.5)	70.6	29.4	0	0	100
4. helps me to formulate appropriate management plan for patients having oromucosal lesions/conditions	3.5(0.6)	52.9	41.2	5.9	0	94.1
5. makes me more confident in diagnosing oromucosal lesions/conditions without additional help	3.5(0.6)	58.8	35.3	5.9	0	94.1
Communication Skills						
6. improves my communication skills with patients having oromucosal lesions/conditions	3.6(0.5)	58.8	41.2	0	0	100
7. makes dentist-patient interaction more interesting	3.4(0.5)	41.2	58.8	0	0	100
8. makes dentist-patient interaction more realistic	3.5(0.5)	52.9	47.1	0	0	100
9. reduces my anxiety when communicating with patients having oromucosal lesions/conditions	3.5(0.5)	47.1	52.9	0	0	100
10. makes me more confident in communicating with patients having oromucosal lesions/conditions without additional help	3.4(0.6)	47.1	47.1	5.9	0	94.1
Impact on Learning						
11. positively enhanced learning of diagnostic and communication skills in Oral Medicine	3.5(0.5)	52.9	47.1	0	0	100
12. increases my willingness in learning diagnostic and communication skills in Oral Medicine	3.5(0.6)	58.8	35.3	5.9	0	94.1
13. makes me more concentrated in learning diagnostic and communication skills in Oral Medicine	3.8(0.4)	76.5	23.5	0	0	100

**Table 4:** Distributions of students’ perceived usefulness of VOMC (*n* = 24).

Question: I think Virtual Oral Medicine Clinic No of Respondents (24)	Mean (SD)	Strongly Agree (%)	Agree (%)	Disagree (%)	Strongly Disagree (%)	Overall Agreement (%)
Diagnostic Skills						
1. improves my diagnostic skills in Oral Medicine	3.1(0.6)	20.0	65.0	15.0	0	85.0
2. helps me to interpret variations in clinical presentation of oromucosal lesions/conditions	3.0(0.7)	35.0	50.0	25.0	0	75.0
3. helps me to diagnose oromucosal lesions/conditions	3.2(0.7)	30.0	55.0	15.0	0	85.0
4. helps me to formulate appropriate management plan for patients having oromucosal lesions/conditions	3.1(0.6)	25.0	65.0	10.0	0	90.0
5. makes me more confident in diagnosing oromucosal lesions/conditions without additional help	2.7(0.7)	5.0	60.0	30.0	5.0	65.0
Communication Skills						
6. improves my communication skills with patients having oromucosal lesions/conditions	2.9(0.6)	5.0	80.0	10.0	5.0	85.0
7. makes dentist-patient interaction more interesting	2.5(0.6)	0	55.0	40.0	5.0	55.0
8. makes dentist-patient interaction more realistic	2.5(0.8)	5.0	50.0	35.0	10.0	55.0
9. reduces my anxiety when communicating with patients having oromucosal lesions/conditions	2.9(0.7)	10.0	70.0	15.0	5.0	80.0
10. makes me more confident in communicating with patients having oromucosal lesions/conditions without additional help	2.8(0.6)	5.0	70.0	20.0	5.0	75.0
Impact on Learning						
11. positively enhanced learning of diagnostic and communication skills in Oral Medicine	3.1(0.5)	15.0	75.0	10.0	0	90.0
12. increases my willingness in learning diagnostic and communication skills in Oral Medicine	3.0(0.6)	20.0	60.0	20.0	0	80.0
13. makes me more concentrated in learning diagnostic and communication skills in Oral Medicine	2.8(0.9)	20.0	40.0	35.0	5.0	60.0

**Table 5:** Comparison of perceived usefulness between control and experimental groups.

Question: I Think Role-Play Activity/Virtual Oral Medicine Clinic	Group	Mean	SD	*p*-Value
Diagnostic Skills				
1. improves my diagnostic skills in Oral Medicine	Control	3.76	0.437	<0.001
	Experimental	3.05	0.605	
2. helps me to interpret variations in clinical presentation of oromucosal lesions/conditions	Control	3.71	0.470	0.003
	Experimental	3.00	0.725	
3. helps me to diagnose oromucosal lesions/conditions	Control	3.71	0.470	0.009
	Experimental	3.15	0.671	
4. helps me to formulate appropriate management plan for patients having oromucosal lesions/conditions	Control	3.47	0.624	0.099
	Experimental	3.15	0.587	
5. makes me more confident in diagnosing oromucosal lesions/conditions without additional help	Control	3.53	0.624	<0.001
	Experimental	2.65	0.671	
Total	Control	18.18	2.351	0.001
	Experimental	15.00	2.555	
Communication Skills				
6. improves my communication skills with patients having oromucosal lesions/conditions	Control	3.59	0.507	<0.001
	Experimental	2.85	0.587	
7. makes dentist-patient interaction more interesting	Control	3.41	0.507	<0.001
	Experimental	2.50	0.607	
8. makes dentist-patient interaction more realistic	Control	3.53	0.514	<0.001
	Experimental	2.50	0761	
9. reduces my anxiety when communicating with patients having oromucosal lesions/conditions	Control	3.47	0.514	0.004
	Experimental	2.85	0.671	
10. makes me more confident in communicating with patients having oromucosal lesions/conditions without additional help	Control	3.41	0.618	0.003
	Experimental	2.75	0.639	
Total	Control	17.41	2.373	0.000
	Experimental	15.00	2.625	
Impact on Learning				
11. positively enhanced learning of diagnostic and communication skills in Oral Medicine	Control	3.53	0.514	0.010
	Experimental	3.05	0.510	
12. increases my willingness in learning diagnostic and communication skills in Oral Medicine	Control	3.53	0.624	0.016
	Experimental	3.00	0.649	
13. makes me more concentrated in learning diagnostic and communication skills in Oral Medicine	Control	3.76	0.437	<0.001
	Experimental	2.75	0.851	
**Total**	Control	10.82	1.286	0.001
	Experimental	8.80	1.824	

## Data Availability

Not applicable.
